# Observational suspected adverse drug reaction profiles of fluoro-pharmaceuticals and potential mimicry of per- and polyfluoroalkyl substances (PFAS) in the United Kingdom

**DOI:** 10.1371/journal.pone.0331286

**Published:** 2025-09-02

**Authors:** Banurja Balasubramaniam, Alan M. Jones

**Affiliations:** School of Pharmacy, School of Health Sciences, College of Medicine and Health, University of Birmingham, Edgbaston, United Kingdom; Makerere University, UGANDA

## Abstract

**Aims:**

The aim of this research is to explore the suspected adverse drug reactions (ADRs) of perfluorinated medicines to determine whether side effects commonly associated with per- and poly-fluoroalkyl substances (PFAS) exposure were correlated to the type or number of fluorine atoms in these medications.

**Methods:**

Thirteen fluorinated drugs and six non-fluorinated (or low fluorinated) comparators were selected after systematic triage. The reported ADR data from the Medicines and Healthcare Products Regulatory Agency’s (MHRA) Yellow Card, and prescribing data from the OpenPrescribing database and the National Health Service Business Service Authority (NHSBSA) over a 5-year period were curated. Prescribing data was used to standardise the ADRs by calculating ADRs/1,000,000 items dispensed for selected system organ classes (SOCs), associated with PFAS exposure, for all 19 drugs. The physiochemical and pharmacological properties of the selected drugs were determined from ChemDraw version 23.1.1, Drug Bank, electronic medicines compendium (EMC) and the chemical database of bioactive molecules with drug-like properties, European Molecular Biology Laboratory (ChEMBL).

**Results:**

Excluding congenital, familial, and genetic disorders, and endocrine disorders, all other SOCs (*n *= 5) showed statistical significance (*P *< .05) for ADRs/1,000,000 items identified across the 13 fluorinated drugs. It was identified that leflunomide was suspected of more ADRs than other comparator medications, which had the highest suspected ADRs/1,000,000 items dispensed (*n *= 343) and lansoprazole had the lowest (*n *= 14). Both drugs contain same number of fluorine atoms (*n *= 3) and similar type of fluorine moiety (trifluoromethyl, -CF_3_).

**Conclusion:**

No correlation between the fluorination status of the drugs and the ADRs were found.

## Introduction

Per-and poly-fluoroalkyl substances (PFAS), often referred to as ‘forever chemicals’ due to their persistence in the environment, bioaccumulate in the body [1] and have been used in consumer products since 1940 [2,3]. Perfluorooctanoic acid (PFOA) and perfluorooctane sulfonic acid (PFOS) are well-known representative examples [4].

PFAS are used in a range of products for their oil and water repellent ability, as well as their resistance to heat. Application of these chemicals range from use in protective coating on clothing, cookware, in agriculture, and packaging, to being used in firefighting foams and electronics [1,3]. Although, they are useful materials, they are not easily eliminated from the body, leading to bioaccumulation [5]. Over time this leads to side effects including cancers, e.g., kidney, testicular, and thyroid cancers [3,6], thyroid problems, e.g., thyroid dysfunction [1,7], liver damage, e.g., non-alcoholic fatty liver disease (NAFLD), liver inflammation [8,9], poor kidney function, e.g., decreased kidney function [10–12], congenital abnormalities/foetal growth, e.g., lower birth weight, increased risk of obesity and higher body mass index (BMI) [13], and neurological effects, e.g., neurodevelopmental issues [14]. Studies identified PFAS (e.g., PFOS) can cross the blood brain barrier (BBB) by disrupting the barrier’s tight junctions and bioaccumulate [15,16]. This supports PFAS’s ability to lead to neurotoxic effects. Whilst these studies give an indication about the impact PFAS has on human health, the full extent remains unelucidated [3].

PFAS are identified using various definitions, ranging from a narrow and focused definition to a broader and more inclusive definition. For instance, TURA (2021b) defines PFAS as substance that contains either a perfluoroalkyl group with ≥ 3 carbon atoms or a perfluoroalkyl ether group with ≥ 2 carbon atoms [2]. The OECD (2021) offers a broader definition by categorising compounds as PFAS if they contain ≥ 1 fully fluorinated methyl or methylene carbon atom [2]. Non-governmental organisations (NGOs) definitions are broader still and include any organic chemical that contains ≥ 1 fluorine atom [2].

Highly fluorinated compounds especially PFAS have raised significant concerns due to their harmful effects on the environment and human health [8]. This has led to all fluorinated compounds being viewed cautiously, including fluorinated drugs [17]. Fluorine is a small atom with high electronegativity that forms a strong carbon-fluorine (C-F) bond. It is widely employed in medicinal chemistry because of its many desirable properties, such as, enhancing metabolic stability, target selectivity and bioavailability [18]. Fluorinated drugs are medications that contain one or more fluorine atoms. These drugs are used across many therapeutic areas and belong to different drug classes. The first fluorinated drug that was discovered in 1954 [19], contained fluorocortisone as the active ingredient and in 1988 its commercialised form, fluorocortisone acetate a corticosteroid, was introduced in the UK [20]. Some well-known fluorine containing drugs includes celecoxib an non-steroidal anti-inflammatory drug (NSAID), fluoxetine an antidepressant, lansoprazole a proton pump inhibitor (PPI), amongst many others [2]. There are over 300 fluorinated drugs that are approved worldwide [2,21].

The growing public awareness to the harms of PFAS and tighter regulation policies [22–24] on them has raised safety questions regarding all fluorinated compounds. Despite fluorinated drugs being structurally distinct from the traditional PFAS molecules, there has been growing apprehension that fluorinated drugs may cause PFAS like adverse drug reactions (ADR) with prolonged exposure. This is due to the parallels between some of the chemical features (e.g., the C-F bond) of fluorinated drugs and PFAS. More recently certain fluorinated drugs are now encompassed by the broader definitions of PFAS. Around 30% of organofluorine drugs fit the OECD 2021 definition and 94% fit the definition of having ≥ 1 fully fluorinated carbon atom [2].

Understanding whether fluorinated drugs are associated with suspected ADRs linked to PFAS exposure can improve drug safety, and may provide insight into long-term effects, and improve patient safety. Although ADRs of fluorinated drugs and the negative health outcomes of PFAS are researched independently at present, [3,7–9,25,26] there is a notable gap in research investigating the connection between fluorinated drugs and ADRs that resemble PFAS health outcomes which this pilot study will address. For context, an ADR is an unintended response to a medication [27]. They are categorised according to Rawlins and Thompson classification as type A, dose dependent and predictable and type B, dose independent and unpredictable [28]. ADRs are common to all medications, they contribute to 16.5% of total hospital admissions and cost the NHS £2.2 Bn *p.a.* in the United Kingdom [29].

### Aims

This research focuses on examining any potential relationship between fluorinated drugs and ADRs associated with heavily fluorinated compounds, such as PFAS and whether the number or type of fluorination present has any influence on the pattern of suspected ADRs reported. The secondary purpose is to explore the drugs’ physiochemical and pharmacological properties to determine if there are any links between these unique descriptors and the observed ADRs of the fluorinated drugs.

## Methods

**Fig 1:**
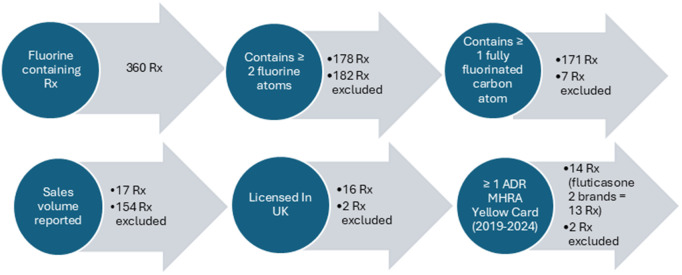
The inclusion/exclusion steps to the selected PFAS-like drugs interrogated in this study.

The list of all approved fluorinated drugs was extracted from Hammel E, et al. (2022) [2]. In total, 13 fluorinated drugs out of 360 were selected for analysis using inclusion and exclusion criteria of this study (**Table 1** and **Fig 1**). The drugs selected were celecoxib, flecainide, fluoxetine, lansoprazole, leflunomide, sitagliptin, travoprost, ezetimibe, fluconazole, fluocinolone, fluticasone, nebivolol and ticagrelor.

**Table 1:** Inclusion and exclusion criteria of the study. Microsoft 365 Excel version 24.11 was used to filter the drugs with ≥ 1 fully fluorinated drugs and ≥ 2 fluorine atoms.

Inclusion criteria	Exclusion criteria
• Contains two or more fluorine atoms • Contains one or more fully fluorinated carbon atom. • Licensed in the UK • ≥ 1 ADRs reported on the Yellow Card iDAPs database between January 2019 and May 2024	• Contains less than two fluorine atoms. • Contains no fully fluorinated carbon atoms. • Not licensed in the UK • No ADRs reported on the Yellow Card iDAPs database between January 2019 and May 2024

### Reported adverse drug reactions data

The data for suspected ADRs reported were collected from Medicines & Healthcare products Regulatory Agency (MHRA) Yellow Card interactive Drug Analysis Profiles (iDAPs) [30]. ADR data were curated from, January 2019 to May 2024 for each of the fluorinated drugs that met the inclusion and exclusion criteria (**Table 1**). The data collection took place in October 2024. Where a structurally similar medication (with fewer or no fluorine atoms) for the same or closely related indication was available (*n *= 6) the respective data was extracted for additional comparison of the effect of fluorine on ADRs.

The system organ classes (SOC) that this study focused on are congenital, familial, and genetic disorders, endocrine disorders, hepatobiliary disorders, neoplasms benign, malignant, and unspecified, nervous system disorders, psychiatric disorders, and renal and urinary disorders.

### Prescribing data

Prescribing data for the 13 selected fluorinated drugs and 6 non-fluorinated or fewer fluorinated comparator drugs were curated from OpenPrescribing [31], from September 2019 to August 2024, for primary care Rx data and NHS Business Services Authority (NHSBSA) [32], from January 2019 to July 2024, for secondary care Rx data. The prescribing data obtained was the number of drugs dispensed in each period. Number of ADRs per 1,000,000 items was then calculated to standardise the data, using the equation: ADRs/1,000,000 items = (Number of ADRs/ Total items dispensed) x 1,000,000 [33].

### Pharmacological interaction

ChEMBL (Chemical database of bioactive molecules with drug-like properties, European Molecular Biology Laboratory) [34] and BindingDB [35] were used to identify the relevant human target proteins and the IC_50_ values associated with them, which indicates the concentration needed to inhibit 50% of the target protein’s activity. The median of the IC_50_ values was calculated where n ≥ 1 IC_50’s_ for a single human target under analogous experimental conditions was identified.

The maximum plasma concentration values (C_max_) of each drug were obtained from DrugBank [36], electronic medicines compendium (EMC) [37] and by searching literature databases (Web of Science and Google Scholar) using the search parameters, drug name and ‘*C*_max_’ and/or ‘maximum plasma concentration’ [38–49]. The reported C_max_ was then converted from ng/ml to nM using the equation: nM = (ng/mL x 1000)/ MW.

The pIC_50_ (-Log_10_(IC_50_)) value was then calculated using the most potent, single target, IC_50_ value gathered from ChEMBL [34] and BindingDB [35], of each drug. This was then used to calculate the lipophilic ligand efficiency (LLE), using the formula LLE = pIC_50_ - cLog_10_P.

### Drug pharmacokinetics and chemical properties

The structure of the drugs was visualised using Chemdraw (version 23.1.1) from Perkin-Elmer ChemOffice. Physiochemical properties of the drugs were curated from ChEMBL [34] and Chemdraw (version 23.1.1). These include, p*K*a, Log P, topological polar surface area (^*t*^PSA), molecular weight (MW), hydrogen bond donors (HBDs), hydrogen bond acceptors (HBA), cLog_10_D and cLog_10_P. DrugBank [36] and the emc [37] were used to extract the pharmacokinetic properties, and the British National Formulary (BNF) [50] was used to extract the dosing regimen. Certain drug properties can increase a drug’s potential to penetrate the blood brain barrier (BBB), which can lead to neurological ADRs [33].

The BBB properties are as follows [33]: MW < 450 Da; HBDs < 6; HBA < 2; ^*t*^PSA < 90 Å^2^; cLog_10_D at pH 7.4 should be between 1–3; Low affinity for p-glycoprotein; p*K*a – neutral or basic. To support the predicted BBB values, the Web of Science search engine was used to find studies regarding cerebrospinal fluid (CSF) penetration values, using the search parameters, drug name and ‘cerebrospinal fluid’ [51–56].

### Statistical analysis

The Chi-squared value of the ADRs in each SOC, across all 13 fluorinated drugs, were calculated using Microsoft 365 Excel version 24.11, to determine the statistical significance of fluorination level vs ADRs observed (*P *< .05 was selected for statistical significance independent of mechanism of action (MoA)) of the suspected ADRs across the drug vs SOC categories. Where ADR reports are *n* = < 5, the data was excluded from the calculation due to statistical uncertainty at low levels of reporting (S1 Appendix) [57].

### Ethical approval

This study did not require School of Pharmacy sub-ethics committee ethical approval, as it used fully anonymised data that are in the public domain.

## Results

### Drug class and fluorination content

All drugs in **Table 2** belong to different drug classes and indications, except for fluticasone and fluocinolone that are both corticosteroids. Sitagliptin and flecainide contains the highest number of fluorine atoms in their structures, with 6 fluorine atoms. Trifluoromethyl (CF_3_) was the common moiety among the drugs, celecoxib, fluoxetine, lansoprazole, leflunomide, travoprost, flecainide and sitagliptin contains at least one CF_3_ in its structure.

**Table 2:** The drug class and the type of fluorine content of each of the fluorinated drugs.

Drug name	Type of Fluorine motif	Drug class	Number of fluorine atoms
**Ezetimibe**	Aryl fluoride x 2	Cholesterol absorption inhibitor	2
**Fluconazole**	Aryl fluoride x 2	Triazole antifungal	2
**Fluocinolone**	Secondary alkyl fluoride; tertiary alkyl fluoride	Corticosteroid	2
**Nebivolol**	Aryl fluoride x2	Selective beta blocking agent	2
**Ticagrelor**	Aryl fluoride x2	Antiplatelet	2
**Celecoxib**	Aryl trifluoromethyl group	NSAID	3
**Fluoxetine**	Aryl trifluoromethyl group	SSRI	3
**Lansoprazole**	Alkyl trifluoromethyl group	Proton pump inhibitor	3
**Leflunomide**	Aryl trifluoromethyl group	DMARD	3
**Travoprost**	Aryl trifluoromethyl group	Prostaglandins and analogues	3
**Fluticasone**	Primary alkyl fluoride, secondary alkyl fluoride	Corticosteroid	3
**Flecainide**	Alkyl trifluoromethyl group x2	Antiarrhythmic, class Ic	6
**Sitagliptin**	Aryl fluoride x2, aryl trifluoromethyl group x1	Dipeptidylpeptidase-4 inhibitor	6
**PFOA**	Alkyl trifluoromethyl group x1, alkyl difluoromethyl group x6	n/a	15

Abbreviations: NSAIDS – Non-steroidal anti-inflammatory drugs; SSRI – Selective serotonin re-uptake inhibitor; DMARDS – Disease-modifying anti-rheumatic drug.

### Physiochemical and pharmacological properties

The physiochemical properties, including properties that enhances the drug’s ability to cross the BBB, and pharmacological properties of the drugs are shown in **Table 3**. During the analysis of the drug’s ability to cross the blood brain barrier (BBB), fluoxetine, leflunomide and nebivolol met 6 out of the 7 BBB penetration properties, suggesting they are more likely to cross the BBB. On the other hand, travoprost and ticagrelor only exhibited 3 out of the 7 BBB penetration properties. None of the drugs had HBA count <2. CSF values for celecoxib, flecainide, leflunomide, sitagliptin and fluconazole were available to compare the BBB predictions.

**Table 3:** Summary of the physiochemical and pharmacological properties of the fluorinated drugs and non- or fewer-fluorinated comparators noted by square brackets.

Drug properties	Celecoxib	Flecainide	Fluoxetine [Atomoxetine]	Lansoprazole [Omeprazole]	Leflunomide	Sitagliptin	Travoprost [Latanoprost]]	Ezetimibe	Fluconazole [Voriconazole]	Fluocinolone [Triamcinolone]	Fluticasone [Beclomethasone]	Nebivolol	Ticagrelor	PFOA
cLog_10_P	4.37	3.66	4.57 [3.94]	2.60 [2.57]	2.32	0.69	3.92 [4.14]	3.96	−0.44 [2.28]	0.75 [0.71]	3.80 [1.24]	3.50	2.55	3.62
*p*IC_50_	7.80	5.64	8.03	6.36	6.30	7.74	4.36	6.43	10.38	8.50	10.12	5.20	6.75	n/a
LLE	3.42	1.98	3.46	3.77	3.98	7.05	0.44	2.47	10.82	7.75	6.32	1.70	4.20	n/a
**BBB penetration properties**														
MW (Da)	381.38	414.35	309.33 [255.16]	369.37 [345.11]	270.21	407.32	500.55 [432.29]	409.43	306.28 [349.12]	412.43 [394.18]	444.52 [408.17]	405.44	522.58	413.97
p*K*a	Neutral	8.96	9.78 [9.81]	Neutral [Neutral]	Neutral	8.17	Neutral [Neutral]	Neutral	Neutral [Neutral]	Neutral [Neutral]	Neutral [Neutral]	8.90	Neutral	−0.64
^t^PSA (Å)	75.76	59.59	21.26 [21.26]	63.05 [72.28]	50.69	74.29	96.22 [86.99]	60.77	76.15 [72.91]	115.06 [115.06]	80.67 [94.83]	70.95	134.63	37.3
HBD	1	2	1 [1]	1 [1]	1	1	3 [3]	2	1 [1]	4 [4]	2 [3]	3	4	1
HBA	4	4	2 [2]	4 [6]	3	5	6 [5]	3	7 [9]	6 [7]	5 [6]	5	11	17
cLog_10_D^7.4^	4.37	1.01	1.83 [1.20]	2.60	2.32	−0.14	3.92	3.96	0.56	0.75	3.80	1.71	2.55	−4.42
P-glycoprotein substrate	Yes	Yes	No	Yes	No	Yes	No	Yes	No	No	Yes	No	Yes	No
Number of BBB properties met	4	5	6	5	6	4	3	4	5	4	4	6	3	4
Cerebrospinal fluid (CSF) penetration	2 ng/mL After single oral dose of 200 mg	0.17 mg/kg	No exact value was reported, but studies indicate CSF penetration in animals like rats [6.6 ng/mL]	[2 µM in dogs]	The active metabolite of leflunomide (teriflunomide) was found at a mean concentration of 68 ng/mL	Study found CSF to plasma ratio of 0.01 in non-human primates	- [-]	–	2.7 mg/L after IV dose of 100 mg/ day for 7 days [0.08–3.93 µg/mL]	- [up to 30 ng/mL]	- [-]	–	–	–
**Pharmacokinetic properties**														
*C*_max_ (nM)	1,841	1,904	4,849 [1,372]	2,835 [2,938]	130 (teriflunomide)	950	0.02 [123]	13	21,940 [1.6]	420 [2.8]	0.3 [1,740]	20	1,766	–
half-life	0.8 - 12 h	20 h	4-6 days [5.2–21 h]	1-2 h [1 h]	2 wks	6.5 h	1.5 h [17 min]	22 h	30 h [6 h]	- [18–36 h]	15.1 h [0.5–2.7 h]	10 h	7 h	2-4 yrs
PPB (%)	97	40	95 [98.7]	97 [95]	99.38	38	- [90]	99.7	11-12 [58]	- [68]	>99 [87]	97.9-98.1	>99	>99.5
Hepatic metabolism	CYP2C9	CYP2D6	CYP2D6 [CYP2D6]	CYP3A4 [CYP2C19]	CYP1A2, 2C19, 3A4	CYP3A4, 2C8	- [CYP3A4]	–	CYP3A4, 2C19, 2C9 [CYP3A4, 2C19, 2C9]	CYP2C9, 3A4, 2C19 {CYP3A4]	CYP3A4 [CYP3A4, CYP3A5]	CYP2D6	CYP3A4	–
Renal excretion	27%	86%	60% [<70%]	14-23% [low]	43%	87%	<2%[major]	11%	93.3% [negligible]	- [major]	1-2% [negligible]	38%	26.5%	–
Acute or Chronic treatment *	Chronic	Chronic	Chronic [Chronic]	Chronic [Chronic]	Chronic	Chronic	Chronic [Chronic]	Chronic	Acute [Acute]	Acute [Acute]	Chronic [Chronic}	Chronic	Chronic	–
Dosing	100 mg BD Or 200 mg OD	50 mg BD, up to 300 mg/ day Or 100 mg BD, max 400 mg/ day	20-60 mg OD [80–100 mg OD or BD]	15 mg OD, up to 180 mg in divided doses [40 mg OD]	Initially 100 mg OD for 3 days, then reduced to 10–20 mg OD	100 mg OD	Apply once daily [Apply once daily]	10 mg OD	150 mg OD Or 150 mg every 3 days for 3 doses then 150 mg OD Or 50 mg OD Or 50-400 mg OD [100–200 mg BD]	Intravitreal injection Topical: apply 1–2 times a day [55–110 µg OD]	Inhalation or nasal spray: 100–500 μg BD Nebulised suspension 100 μg OD Topical: apply 1–2 times [40–80 µg OD or BD puffs]	2.5-5 mg OD. Elderly: initially 1.25 mg	Initially 180 mg OD, then 90 mg BD up to 12 months Or 60 mg BD	–

*: Certain drugs are used for chronic and acute treatments, but this table shows what type of treatment they are used from in the majority of cases. Dash (-): Data not available. The values highlighted in grey meet the BBB thresholds. Abbreviations: cLog_10_P - calculated partition coefficient; LLE – lipophilic ligand efficiency; MW – Molecular weight; p*K*a – acid dissociation constant; ^*t*^PSA - topological polar surface area; HBD – Hydrogen bond donor(s); HBA – Hydrogen bond acceptor(s); *C*_max_ - peak serum concentration; PPB – plasma protein binding; h – hour(s); OD – once daily; BD – twice daily.

Fluoxetine was found to be the most lipophilic drug (cLog_10_P value of 4.57) followed by celecoxib with cLog_10_P of 4.37. This suggests high potential to permeate biological membranes.

LLE value of >5 suggests reduced risk of toxicity from promiscuous pharmacological interactions. All drugs except sitagliptin, fluconazole, fluocinolone and fluticasone have an LLE value <5 [58], with travoprost having the lowest LLE of 0.4 and fluconazole with the highest LLE of 10.8. In addition, fluconazole and fluocinolone are the only two drugs among the 13 fluorinated drugs that are primarily used acutely, while the remaining drugs are typically used for chronic treatment (defined as over 3 months [59]).

A comparison between an exemplar PFAS (PFOA) revealed key similarities between the fluorinated medications (LogP 3.62 vs 2.78 and MW 413.97 vs 396.4, average values respectively) but also key differences (acidic nature vs neutral or basic drugs, almost complete PPB, level of fluorination (15 vs ≤ 6 fluorine atoms, respectively), and elongated half-life years vs minute to hours, respectively). Side-by-side comparison of fluorinated vs non-fluorinated drugs revealed an expected increase in physiological half-life of the fluorinated analogue.

### Target affinity

Celecoxib and fluoxetine, whose primary mechanism is the inhibition of the COX-2 enzyme [38] and serotonin receptors respectively, demonstrated the most potent off-target activity (*n* = 12 and 13 pharmacological interactions, respectively). This indicates that the drug can interact with off-target sites at concentrations sufficient to effect their function, as their IC_50_ values are lower than the drug’s maximum plasma concentration (*C*_max_). In contrast, none of the IC_50_ values for flecainide, leflunomide, travoprost, ezetimibe, and nebivolol were below the studied *C*_max_ values. 

### Adverse drug reactions

**Table 5** presents the data of ADRs reported for selected SOCs from 01/2019–05/2024 along with the Chi-squared statistical analysis value (*P*-value) and standardised data of ADRs per million items dispensed, using the total items dispensed for each drug from 08/2019–07/2024 (primary and secondary care). The most prescribed drug was lansoprazole with 315,918,768 items dispensed, followed by fluoxetine with 72,023,468 items. The least prescribed drug was fluocinolone, with 790,377 items. Overall, lansoprazole had the highest total number of ADRs reported for the fluorinated drugs studied (*n* = 4,444), however, it was leflunomide that had the highest number of total ADRs per 1,000,000 items dispensed (*n *= 343). Leflunomide is an immunomodulatory drug used medically to treat rheumatoid and psoriatic arthritis via inhibiting the enzyme dihydroorotate dehydrogenase (DHODH). The majority of the ADRs associated with leflunomide are associated with SOCs outside the scope of PFAS-related side effects, e.g., gastrointestinal, and skin and subcutaneous tissue disorders.

**Table 5:** Summary of the total ADRs reported for selected system organ classes (SOCs) that are associated with PFAS ADRs versus a non-fluorinated (or fewer fluorinated) comparator where available, the standardised total ADRs per million prescriptions, and *P*-values.

	Celecoxib	Flecainide	Fluoxetine [Atomoxetine]	Lansoprazole [Omeprazole]	Leflunomide	Sitagliptin	Travoprost [Latanoprost]	Ezetimibe	Fluconazole [voriconazole*]	Fluocinolone [triamcinolone*]	Fluticasone [Beclomethasone]	Nebivolol	Ticagrelor	*P*
**Total prescriptions**	3,513,088	4,451,832	72,023,468 [30,357,498]	315,918,768 [176,640,208]	1,879,256	23,128,919	3,332,629 [14,970,264]	24,453,776	5,290,067 [2304]	790,377 [1,157,176]	46,739,343 [72,493,252]	6,076,689	5,543,070	**–**
**Congenital, familial, and genetic disorders ADRs** **(ADRs/ 1,000,000 items)**	0 (0)	1 (0.2)	9 (0.1) [0 (0.0)]	7 (0.02) [4 (0.02)	2 (1.1)	0 (0)	0 (0) [0 (0.0)]	1 (0.04)	0 (0) [0(0.0)]	0 (0.00) [0 (0.0)]	1 (0.02) [1 (0.01)]	0 (0)	0 (0)	0.79
**Fatalities** **(Fatalities/ 1,000,000 items)**	0 (0)	0 (0.0)	1 (0.01) [0 (0.0)]	0 (0) [0 (0.0)]	0 (0)	0 (0)	0 (0) [0 (0.0)]	0 (0)	0 (0) [0(0.0)]	0 (0.00) [0 (0.0)]	0 (0) [0 (0.0)]	0 (0)	0 (0)	–
**Endocrine disorders ADRs** **(ADRs/ 1,000,000 items)**	0 (0)	0 (0)	5 (0.1) [0 (0.0)]	24 (0.1) [17 (0.1)]	0 (0)	0 (0)	0 (0) [0 (0.0)]	1 (0.04)	1 (0.2) [1 (434)]	3 (3.8) [9 (7.8)]	29 (0.6) [11 (0.2)]	0 (0)	0 (0)	0.68
**Fatalities** **(Fatalities/ 1,000,000 items)**	0 (0)	0 (0)	0 (0) [0 (0.0)]	0 (0) [0 (0.0)]	0 (0)	0 (0)	0 (0) [0 (0.0)]	0 (0)	0 (0) [0 (0.0)]	0 (0) [0 (0.0)]	0 (0) [0 (0.0)]	0 (0)	0 (0)	–
**Hepatobiliary disorders ADRs** **(ADRs/ 1,000,000 items)**	4 (1.1)	2 (0.5)	7 (0.1) [22 (0.7)]	28 (0.1) [28 (0.2)]	24 (12.8)	1 (0.04)	0 (0) [0 (0.0)]	7 (0.3)	8 (1.5) [13 (5,642)]	0 (0) [1 (0.9)]	0 (0) [0 (0.0)]	0 (0)	1 (0.2)	*P *< .05
**Fatalities** **(Fatalities/ 1,000,000 items)**	0 (0)	0 (0)	0 (0) [0 (0.0)]	2 (0.01) [2 (0.01)]	2 (1.1)	1 (0.04)	0 (0) [0 (0.0)]	0 (0)	0 (0) [0 (0)]	0 (0) [0 (0.0)]	0 (0) [0 (0.0)]	0 (0)	0 (0)	–
**Neoplasms benign, malignant, and unspecified ADRs** **(ADRs/ 1,000,000 items)**	0 (0)	0 (0)	11 (0.2) [0 (0.0)]	8 (0.03) [26 (0.2)	10 (5.3)	3 (0.1)	0 (0) [0 (0.0)]	2 (0.1)	2 (0.4) [33 (14,323)]	0 (0) [0 (0.0)]	0 (0) [2 (0.02)]	0 (0)	4 (0.7)	*P *< .05
**Fatalities** **(Fatalities/ 1,000,000 items)**	0 (0)	0 (0)	0 (0) [0 (0.0)]	1 (0.003) [5 (0.03)]	0 (0)	0 (0)	0 (0) [0 (0.0)]	0 (0)	0 (0) [3 (1,302)]	0 (0) [0 (0.0)]	0 (0) [2 (0.02)]	0 (0)	0 (0)	–
**Nervous system disorders ADRs** **(ADRs/ 1,000,000 items)**	20 (5.7)	31 (7.0)	437 (6.1) [71 (2.3)]	504 (1.6) [602 (3.4)]	41 (21.8)	59 (2.6)	4 (1.2) [98 (6.5)]	103 (4.2)	88 (16.6) [33 (14,323)]	12 (15.2) [94 (81.0)	110 (2.4) [659 (9.1)]	14 (2.3)	51 (9.2)	*P *< .05
**Fatalities** **(Fatalities/ 1,000,000 items)**	1 (0.2)	0 (0)	0 (0) [1 (0.03)]	0 (0) [3 (0.02)]	0 (0)	0 (0)	0 (0) [0 (0.0)]	0 (0)	0 (0) [0 (0.0)]	0 (0.0) [0 (0.0)]	0 (0.00) [0 (0.0)]	0 (0.00)	2 (0.36)	–
**Psychiatric disorders ADRs** **(ADRs/ 1,000,000 items)**	6 (1.7)	12 (2.7)	601 (8.3) [125 (4.1)]	234 (0.7) [412 (2.3)]	19 (10.1)	7 (0.3)	2 (0.6) [19 (1.3)]	47 (1.9)	29 (5.5) [26 (11,285)]	20 (25.3) [52 (44.8)]	81 (1.7) [325 (4.5)]	8 (1.32)	20 (3.61)	*P *< .05
**Fatalities** **(Fatalities/ 1,000,000 items)**	0 (0)	0 (0)	4 (0.1) [0 (0.0)]	0 (0) [1 (0.01)]	0 (0)	0 (0)	0 (0) [0 (0.0)]	0 (0)	0 (0) [0 (0.0)]	0 (0.0) [0 (0.0)]	0 (0.00) [0 (0.0)]	0 (0)	0 (0)	–
**Renal and urinary disorders ADRs** **(ADRs/ 1,000,000 items)**	5 (1.4)	2 (0.5)	17 (0.2) [11 (0.4)]	75 (0.2) [150 (0.9)]	23 (12.2)	7 (0.3)	2 (0.6) [3 (0.2)]	11 (0.5)	11 (2.1) [13 (5,642)]	0 (0.0) [4 (3.4)]	7 (0.2) [14 (0.2)]	0 (0.00)	18 (3.3)	*P *< .05
**Fatalities** **(Fatalities/ 1,000,000 items)**	0 (0)	1 (0.2)	0 (0) [0 (0.0)]	1 (0.003) [1 (0.01)]	1 (0.5)	0 (0)	0 (0) [0 (0.0)]	0 (0)	0 (0) [0 (0.0)]	0 (0.0) [0 (0.0)]	0 (0.00) [0 (0.0)]	0 (0.00)	0 (0.0)	–
**Total ADRs** **(ADRs/ 1,000,000 items)**	210 (59.8)	409 (91.9)	2687 (37.3) [488 (16.1)]	4444 (14.1) [5899 (33.4)]	644 (342.7)	417 (18.0)	57 (17.1) [961 (665)]	939 (38.4)	1096 (207.2) [527 (228,733)]	235 (297) [859 (741)]	940 (20.1) [5689 (78.5)]	105 (17.3)	539 (97.2)	*P *< .05
**Total fatalities** **(Fatalities/ 1,000,000 items)**	1 (0.3)	4 (0.9)	24 (0.3) [1 (0.03)]	22 (0.1) [39 (0.2)]	8 (4.3)	7 (0.3)	0 (0) [2 (0.1)]	1 (0.04)	17 (3.2) [30 (13,021)]	0 (0) [0 (0.0)]	1 (0.02) [9 (0.1)]	0 (0)	6 (1.1)	–

Brackets () – The values inside the brackets are the standardised values, ADRs/1,000,000 items dispensed and/or fatalities/1,000,000 items dispensed. Square brackets indicate a close structural analogue without PFAS properties for a similar MoA (source: https://www.cheminfo.org/Chemistry/Database/DrugBank/Structure_search/index.html). * Voriconazole contains one fluorine atom and is structurally similar and similar indication.

### ADRs associated with perfluorinated forever chemicals

Excluding congenital, familial, genetic, and endocrine disorders, statistical significance (*P *< .05) was observed for all other SOCs studied. The highest number of ADRs/1,000,000 items for congenital, familial, and genetic disorders ADRs was reported for leflunomide (1.1 ADRs/1,000,000 items). The reported ADRs include cardiac and vascular disorders (congenital), and congenital and hereditary disorders not elsewhere classified (NEC) [30]. Leflunomide also had the highest ADRs reported per 1,000,000 items for hepatobiliary disorders, neoplasms benign, malignant, and unspecified, nervous system disorders and renal and urinary disorders, with 12.8, 5.3, 21.8, and 12.2 ADRs/1,000,000 items, respectively. The majority of the studied drugs had no ADRs reported for endocrine disorders. The highest number of endocrine disorders were seen in fluocinolone with 3.8 ADRs/1,000,000 items and fluticasone with 0.6 ADRs/1,000,000 items. Fluocinolone is a corticosteroid for severe inflammatory disorders including eczema (unresponsive to less potent corticosteroids) and psoriasis acting upon the glucocorticoid receptor. The majority of the listed ADRs are associated with eye, and skin and subcutaneous tissue disorders which are outside the scope of PFAS side effects. The higher number of ADRs associated with fluocinolone is due to both a smaller amount of Rx and a small number of endocrine reports (*n *= 3).

For psychiatric disorders, the highest ADR number was seen in fluocinolone with 25.3 ADRs/1,000,000 items. The majority of those ADRs were reported for anxiety disorders and symptoms (*n* = 11 cases) [30].

## Discussion

The 13 fluorinated drugs, except fluticasone and fluocinolone which both belong to the corticosteroid drug class, belongs to different drug classes, meaning their MoA and clinical indication differs from each other. This indicates that the drugs are likely to have different pharmacological and side effect profiles. The majority of pharmacological interactions from **Table 4**, the IC_50_ values exceeds their *C*_max_ values, are unlikely to play a significant role in a clinical setting, unless there were drug bioaccumulation in operation, which is known to occur with leflunomide [42].

**Table 4:** Summary of the fluorinated drugs’ interaction with different target proteins as median IC_50_ values and the drugs’ *C*_max_ values. Non-fluorinated or fewer fluorinated drug comparators are denoted by square brackets.

Target (nM)	Celecoxib	Flecainide	Fluoxetine [Atomoxetine]	Lansoprazole [Omeprazole]	Leflunomide	Sitagliptin	Travoprost [Latanoprost]	Ezetimibe	Fluconazole [Voriconazole]	Fluocinolone [Triamcinolone]	Fluticasone [Beclomethasone]	Nebivolol	Ticagrelor
Cyclooxygenase-1	14,000	–	–	–	–	–	–	–	–	–	–	–	–
Cyclooxygenase-2	**90**	–	–	–	–	–	–	–	–	–	–	–	–
3-phosphoinositide dependent protein kinase-1	48,000	–	**130**	–	–	–	–	–	–	–	–	–	–
Adenosine A3 receptor	24,622	–	–	–	–	–	–	–	–	–	–	–	–
Alpha-2b adrenergic receptor	**1,516**	–	**1,528**	–	–	–	–	–	–	–	–	–	–
Angiotensin-converting enzyme	–	–	–	–	–	11,000	–	–	–	–	–	–	–
Arachidonate 15-lipoxygenase	**49**	–	–	–	–	–	–	–	–	–	–	–	–
Arachidonate 5-lipoxygenase	**1,720**	–	–	–	–	–	–	–	–	–	–	–	–
ATP-binding cassette sub-family G member 2	–	–	–	14,400	–	–	–	–	–	–	–	–	–
Beta-3 adrenergic receptor	16,124	–	–	–	–	–	–	–	–	–	–	–	–
Bile salt export pump (BSEP)	–	217,600	143,800	–	143,700	147,500	43,900 [12,900]	56,000	–	–	- [10,300]	–	–
Carbonic anhydrase I	50,000	–	–	–	–	–	–	–	–	–	–	–	–
Carbonic anhydrase II	**21**	–	–	–	–	–	–	–	–	–	–	–	–
Carbonic anhydrase IX	**16**	–	–	–	–	–	–	–	–	–	–	–	–
Cytochrome c oxidase subunit 2	**293**	–	–	–	–	–	–	–	–	–	–	–	–
Cytochrome P450 11B1	–	–	–	–	–	–	–	–	30,000	–	–	–	–
Cytochrome P450 11B2	–	–	–	–	–	–	–	–	**13,000**	–	–	–	–
Cytochrome P450 19A1	–	–	–	–	–	–	–	–	**22,000**	–	–	–	–
Cytochrome P450 1A2	–	–	–	6,460	500	–	–	–	–	–	–	–	–
Cytochrome P450 1B1	–	–	475000	–	–	–	–	–	–	–	–	–	–
Cytochrome P450 2C19	–	–	**53**	6,868	–	–	–	–	**8,200** [8,700]	–	–	–	–
Cytochrome P450 2C8	–	–	–	5,750	–	–	–	–	–	–	–	–	–
Cytochrome P450 2C9	10,000	–	–	19,200	–	–	–	–	29,000 [8,400]	–	–	–	–
Cytochrome P450 2D6	**1,000**	–	**700** [2,000]	4,107	–	–	–	–	–	–	–	–	–
Cytochrome P450 3A4	–	–	–	- [77,983]	–	–	–	–	**16,985** [2,500]	–	–	–	–
Cytochrome P450 3A5	–	–	–	–	–	–	–	–	**8,000**	–	–	–	–
Cytochrome P450 46A1	–	–	–	–	–	–	–	–	- [35]	–	–	–	–
Cytochrome P450 51	–	–	–	–	–	–	–	–	**0.04**	–	–	–	–
DFDOH	–	–	–	–	1,250	–	–	–	**–**	–	–	–	–
Dihydroorotate dehydrogenase	–	–	–	–	11,500	–	–	–	–	–	–	–	–
Dipeptidyl peptidase II	–	–	–	–	–	59,830	–	–	–	–	–	–	–
Dipeptidyl peptidase IV	–	–	–	–	–	**18**	–	–	–	–	–	–	–
Dipeptidyl peptidase IX	–	–	–	–	–	67,410	–	–	–	–	–	–	–
Dipeptidyl peptidase VIII	–	–	–	–	–	48,000	–	–	–	–	–	–	–
Dopamine D1 receptor	27,994	–	–	–	–	–	–	–	–	–	–	–	–
Dopamine transporter	2,431	–	13,760 [3,100]	–	2,884	–	–	–	–	–	–	–	–
Endo-β-*N*-acetylglucosaminidase	–	–	–	24,380	–	–	–	–	–	–	–	–	–
Epoxide hydratase	131,070	–	–	–	–	–	–	–	–	–	–	–	–
Fatty acid synthase	–	–	–	93,000	–	–	–	–	–	–	–	–	–
Fibroblast activation protein alpha	–	–	–	–	–	30,390	–	–	–	–	–	–	–
Glucocorticoid receptor	–	–	–	–	–	–	–	–	–	**3**	**0.1**	–	–
HERG	–	3890	**1,514**	–	–	501,187	–	–	–	–	–	6,310	–
Histone deacetylase 1	**1,185**	–	–	–	–	–	–	–	–	–	–	–	–
Histone deacetylase 6	**643**	–	–	–	–	–	–	–	–	–	–	–	–
MAP kinase p38 alpha	**810**	–	–	–	–	–	–	–	–	–	–	–	–
Monoamine oxidase A	–	–	–	–	20,696	–	–	–	–	–	–	–	–
Multidrug resistance-associated protein 4	–	–	–	–	–	–	74,000	–	–	–	–	–	–
Muscarinic acetylcholine receptor M1	–	–	**3,203**	–	–	–	–	–	–	–	–	–	–
Muscarinic acetylcholine receptor M3	–	–	**3,595**	–	–	–	–	–	–	–	–	–	–
Muscarinic acetylcholine receptor M5	–	–	**1,358**	–	–	–	–	–	–	–	–	–	–
N(G),N(G)-dimethylarginine dimethylaminohydrolase 1	–	–	–	51,000	–	–	–	–	–	–	–	–	–
Niemann-Pick C1-like protein 1	–	–	–	–	–	–	–	370	–	–	–	–	–
Norepinephrine transporter (NET)	7,308	2,268	- **[5]**	4,630	–	–	–	–	–	–	–	–	–
P-glycoprotein 1	–	–	115,500	62,800	–	–	–	–	–	–	–	–	–
Phosphodiesterase 5A	36,500	–	–		–	–	–	–	–	–	–	–	–
Phosphoethanolamine/phosphocholine phosphatase	–	–	–	**434**	–	–	–	–	–	–	–	–	–
Potassium channel subfamily K member 2	–	–	16,500	–	–	–	–	–	–	–	–	–	–
Prostaglandin E synthase	**540**	–	–	–	–	–	–	–	–	–	–	–	–
Protein-arginine deiminase type-4	–	–	–	–	2,400,000	–	–	–	–	–	–	–	–
Serotonin 2a (5-HT2a) receptor	–	–	**452**	–	–	–	–	–	–	–	–	–	–
Serotonin 2c (5-HT2c) receptor	–	–	**140**	–	–	–	–	–	–	–	–	–	–
Serotonin 6 (5-HT6) receptor	–	–	**1,661**	–	–	–	–	–	–	–	–	–	–
Serotonin transporter	6,276	–	**9 [190]**	–	–	–	–	–	–	–	–	–	–
Sigma opioid receptor	–	–	**675**	–	–	–	–	–	–	–	–	–	–
Sodium channel protein type V alpha subunit	–	6,500	–	–	–	712,643	–	–	–	–	–	7,126	–
Voltage-gated potassium channel subunit Kv1.1	–	209,000	–	–	–	–	–	–	–	–	–	–	–
Voltage-gated potassium channel subunit Kv1.5	–	51,000	–	–	–	–	–	–	–	–	–	–	–
Voltage-gated potassium channel subunit Kv4.3	–	–	–	–	–	100,000,000	–	–	–	–	–	50,119	–
Voltage-gated L-type calcium channel alpha-1C subunit	–	–	–	–	–	100,000,000	–	–	–	–	–	15,849	–
P2Y purinoceptor 12	–	–	–	–	–	–	–	–	–	–	–	–	**180**
P2Y purinoceptor 13	–	–	–	–	–	–	–	–	–	–	–	–	**1,400**
TSC22 domain family protein 3	–	–	–	–	–	–	–	–	–	–	**0.2**	–	–
Progesterone receptor	–	–	–	–	–	–	–	–	–	–	21	–	–
Mineralocorticoid receptor	–	–	–	–	–	–	–	–	–	–	149	–	–
***C***_**max**_ **(nM)**	1,841	1,904	4,849 [1,372]	2,835 [2,928]	130 (as teriflunomide)	950	0.02 [123]	13	21,940 [1.6]	420 [2.8]	0.3 [1,740]	20	1,766

Dash (-) = The value is either not determined or not reported. **Bold** the clinically achievable inhibitory concentrations versus respective C_max_. NET = Norepinephrine transporter.

The highest number of suspected ADRs reported for lansoprazole can be attributed to it being the most prescribed drug amongst all drugs studied, ~ 400 times more than the least prescribed drug fluocinolone. However, lansoprazole has the lowest total ADRs rate of 14.1 ADRs/1,000,000 items.

### Fluorine content and ADR pattern

From inspection of **Tables 2** and **5**, the drugs analysed revealed no clear relationship between the variability observed in the ADRs reported and the (number of and/or type of) fluorination of the drugs. The difference in the number of fluorine or the type of fluorine moieties the drug contains does not appear to correlate with the reported ADR incidences. Moreover, the ADR pattern is due to the underlying MoA, indication, and potential associated confounders.

For instance, the drugs sitagliptin and flecainide, containing the highest number of fluorine atoms (*n* = 6) among the 13 drugs studied, but did not exhibit the highest ADR rate in any of the selected SOCs. In contrast, leflunomide, with only 3 fluorine atoms showed significantly higher ADR rates in multiple SOCs, including congenital, familial, and genetic disorders, hepatobiliary, neoplasms, nervous system, and renal disorders with 1.1, 12.8, 5.3, 21.8 and 12.2 ADRs/1,000,000 items, respectively. One explanation for this could be leflunomide’s long half-life of 2 weeks, meaning it remains in the body longer compared to other drugs. While its prolonged exposure supports its therapeutic use, it also means that it can lead to drug [42] accumulation and increase the possibility of ADRs resulting.

The type of fluorine moiety also has no clear influence on ADR rates, as drugs with same fluorine moiety had distinct ADRs profiles. For example, leflunomide and lansoprazole, both containing 3 fluorine atoms via a trifluoromethyl group (CF_3_), a functional group that is commonly present in both per-and poly fluorinated compounds, exhibited significantly different ADR rates. Leflunomide reported the highest ADR rate at 342.7 ADRs/1,000,000 items, while lansoprazole reported the lowest at 14.1 ADRs/1,000,000 items. Similarly, ezetimibe and nebivolol, both containing a fluorophenyl group, showed notable differences in ADR rates. Ezetimibe’s total ADR rate, 38.4 ADRs/1,000,000 items, was double that of nebivolol’s, 17.3 ADRs/1,000,000 items. Moreover, ezetimibe had ADRs reported across all selected SOCs, whereas nebivolol had ADRs in only 2 of the selected SOCs, which were nervous system and psychiatric disorders.

For the six fluorinated drugs with a structurally similar non-fluorinated or fewer fluorinated comparator for the same indication that were available the following trends emerged: (1) Fluoxetine vs atomoxetine – In all SOCs the fluorinated drug had a higher ADR rate except for hepatobiliary and renal and urinary disorders; (2) Lansoprazole vs omeprazole – In all SOCs the non-fluorinated drug had higher ADR rate except for congenital, familial, and genetic disorders, and endocrine disorders where the ADRs were comparable; (3) Travoprost vs latanoprost – Where data was available the non-fluorinated drug had higher ADR rates (nervous system disorders, psychiatric disorders) apart from renal and urinary disorders; (4) Fluconazole vs voriconazole – in all cases the fewer fluorine atom containing voriconazole had higher ADR rates (endocrine disorders, hepatobiliary disorders, neoplasms benign, malignant, and unspecified ADRs, nervous system disorders, and psychiatric disorders); (5) Fluocinolone vs triamcinolone – in all cases triamcinolone had higher ADR rates (endocrine disorders, hepatobiliary disorders, nervous system disorders, psychiatric disorders, and renal and urinary disorders); and (6) Fluticasone vs beclomethasone – apart from congenital, familial, and genetic disorders, and endocrine disorders, the non-fluorinated comparator had higher ADR rates (neoplasms benign, malignant, and unspecified ADRs, nervous system disorders, and psychiatric disorders). Further supporting the premise that fluorination status does not give rise to a significant uptick in ADRs commonly associated with PFAS.

### ADRs associated with fluorinated drugs

#### Congenital, familial, and genetic disorders.

Congenital, familial, and genetic disorders ADRs reported are low across all studied drugs compared to other SOCs (**Table 5**), with leflunomide, a disease modifying anti-rheumatic drug (DMARD), reporting the highest rate at 1.1 ADRs/1,000,000 items. This effect may be attributed to teriflunomide, the active metabolite of leflunomide, which inhibits dihydroorotate dehydrogenase (DHODH) (**Table 4**) in the *de novo* pyrimidine synthesis pathway [60]. The teratogenic potential of leflunomide might stem from this metabolites’ inhibition rather than fluorine content [61]. Moreover, leflunomide is a drug that is most used by females [62] as rheumatoid arthritis (RA), an indication treated by leflunomide, is more prevalent in women [63]. The peak onset of RA is also between the ages 30–40 [63], which is within the typical childbearing age range [64]. A study also found that women report more ADRs than men [65]. These factors may have influenced the high ADR rate of leflunomide in this category.

Currently, evidence that suggests leflunomide can cause congenital disorders in humans are lacking, however teratogenicity has been reported in animals including rabbits and rats [62,66,67]. A study found that leflunomide in mice can lead to anencephaly, weight reduction in the exposed embryos, malformations, and haemorrhages [62]. This also aligns with the advice on BNF, that suggest avoiding leflunomide in pregnant and breastfeeding women and wait up to 2 years for women and 3 months for men before conception or consider a washout procedure [42,68].

#### Endocrine disorders.

Perfluorinated chemicals like PFAS are known to cause endocrine problems, especially, thyroid problems [1,7]. Whilst fluocinolone and fluticasone exhibited the two highest endocrine disorders ADRs/1,000,000 items, 3.8 and 0.6 respectively, both drugs have no thyroid gland related reports (*n* = 0). On the other hand, lansoprazole, and ezetimibe, which has two of the lowest endocrine disorder ADRs/1,000,000 items, 0.1 and 0.04 respectively, exhibited ADRs associated with thyroid gland disorders, with *n *= 3 and *n = *1 reported cases, respectively.

Although thyroid-related ADRs were noted for two of the drugs, the small number of reports suggests these occurrences are rare. Additionally, the *P*-value for the endocrine SOC was 0.68 (*P *> .05), indicating no statistical significance.

Lansoprazole, a proton pump inhibitor (PPI), is known to cause hypomagnesemia [69], which has been linked to hyperparathyroidism. Studies indicate patients with hypomagnesemia exhibited more severe hyperthyroidism compared to those without [70] and it can also trigger secondary hyperparathyroidism [71]. Moreover, lansoprazole can interact with levothyroxine [72], a common thyroid medication. If a patient is on levothyroxine and is subsequently prescribed lansoprazole, the PPI can reduce levothyroxine absorption and potentially cause poorly controlled thyroid function [72]. However, hypomagnesemia and interaction with levothyroxine were also seen in other PPIs, including those without any fluorine atoms in their structure, such as omeprazole [73–76]. This is further supported, as *n *= 6 thyroid ADRs were made for omeprazole in the same time period (01/2019-05/2024) [30]. Ezetimibe only had *n *= 1 report of thyroid gland disorder.

#### Hepatobiliary disorders.

Leflunomide (12.8 ADRs/1,000,000 items) presented the highest ADRs/1,000,000 items for hepatobiliary disorders. Notably leflunomide interacts with bile salt export pump (BSEP) (**Table 4**), which facilitates the secretion of bile salt, a process important to maintain the bile flow and enterohepatic recirculation of bile salt [77]. This can lead to drug induced liver injury (DILI) [78]. The BNF also advises caution regarding hepatotoxicity when using leflunomide and to monitor liver function for any abnormalities. If an abnormality is found, then the drug dose is reduced or discontinued according to the results [68]. However, hepatotoxicity is not limited to leflunomide, other non-fluorinated DMARDs can also be associated with this ADR [79].

#### Neoplasms benign, malignant, and unspecified.

PFAS in the bloodstream was found to alter the glycosylation process of immunoglobulin G (IgG) leading to an impact on the immune system [3]. This leads to increased cancer risk, as glycosylation of IgG linked to development of disease [3]. Exposure to PFAS, particularly perfluorooctanoic acid (PFOA), has been linked to kidney and testicular cancer [80].

Leflunomide, which also acts on the immune system and has an immunosuppression effect [42], had the highest ADR/1,000,000 items (5.3). However, it is attributed to contribute to anti-cancer effects [81,82]. Furthermore, no kidney or testicular cancer-related ADRs were reported [30].

A study which explored leflunomide’s effects on melanoma found that the use of leflunomide in combination with selumetinib improved tumour size reduction *in vivo* [82]. This evidence does not align with the suspected ADRs reported on MHRA Yellow Card database, as the majority of the ADRs reported under neoplasm were skin neoplasm [30]. One explanation for this would be that the reported ADRs were a result of the confounding disease indication and not the drug leflunomide. Rheumatoid arthritis (RA), an indication for leflunomide, can increase the risk of cancers [83]. RA was found to increase the risk of non-melanoma skin cancer (NMSC) and leflunomide was found to have no association [84].

#### Nervous system and psychiatric disorders.

Nervous system disorders were reported for all 13 drugs and is one of the frequently reported ADR across all studied drugs. Based on the BBB penetration properties and the concentrations found in CSF, leflunomide, fluoxetine and nebivolol were expected to have the highest ADRs/1,000,000 items, as they all met 6 out the 7 BBB penetration properties. Moreover, ~ 68ng/mL of leflunomide’s major metabolite (teriflunomide) was found in CSF [54], whereas no CSF concentration data for fluoxetine and nebivolol in humans were available for comparison.

As expected, leflunomide had the highest ADR rates with 21.8 ADRs/1,000,000 items. However, nebivolol and fluoxetine, exhibited low ADR rates compared to leflunomide. Nebivolol has 2.3 ADRs/1,000,000 items, approximately 10-fold lower than leflunomide’s, and fluoxetine with 6.1 ADRs/1,000,000 items, which is approximately 3-fold lower.

Psychiatric disorder ADRs follow the same prediction as nervous system disorder using the BBB penetrability score and concentration in CSF. However, fluocinolone was identified to exhibit the highest ADR rate with 25.3 ADRs/1,000,000 items followed by the predicted drugs leflunomide (10.1 ADRs/1,000,000 items) then fluoxetine (8.3 ADRs/1,000,000 items).

The underlying mechanism for fluocinolone’s suspected psychiatric ADR profile remains unelucidated, but a plausible reason for its high ADR rate might be the route of administration. It is given as an intravitreal injection [85], a procedure that can cause anxiety, which was the most reported ADR [30] by patients [86].

Peripheral neuropathy (PN) is listed in the BNF as a common, and anxiety as an uncommon, side effect for leflunomide [68]. This data supports that leflunomide is more likely to be associated with nervous system disorders compared to psychiatric disorders. Leflunomide’s interaction with dopamine receptors (**Table 4**), is a potential avenue to help explain the PN, as dopamine transporters contribute to Parkinson’s disease (PD) [87] and PN is more prevalent in patients with PD [88].

The BNF states fluoxetine can cause various psychiatric disorders, for example anxiety, confusion, hallucination, and suicidal behaviours [89]. Fluoxetine also interacts with dopamine transporter along with serotonin receptors and transporters (**Table 4**).

#### Renal and urinary disorders.

Increased renal clearance is associated with higher renal ADRs [90], as it makes the kidneys likely to be more exposed to the drugs or its active metabolite. The active metabolite of leflunomide, the drug that has the highest ADR incidence of 12.2 ADRs/1,000,000 items, is cleared renally alongside hepatic clearance (**Table 3**) [42]. Renal failure was the most reported suspected ADR (*n* = 12 cases) [30]. The exact mechanism for this is not fully understood, but studies support these findings [91,92]. It should be noted that other DMARDs also possess potential to be associated with nephrotoxicity [79].

### Strengths of the Study

This study adds to the body of knowledge of pharmacovigilance with an approach that considers the concerning PFAS-like nature of fluorinated molecules that meet the inclusion criteria. A critical appraisal that integrates prescribing patterns, ADRs, physicochemical, pharmacokinetics, and off-target pharmacology is employed to understand whether ADRs are truly associated with fluorination status or are the result of confounding effects.

### Limitations

The ADRs reported through the Yellow Card scheme are suspected and therefore do not require confirmed causal relationship prior to submission. One of the common limitations is underreporting of suspected ADRs leading to potential underestimation of the ADRs. A systematic review found the median underreporting to be 94% [93]. A major cause of this is lack of knowledge [94]. This can lead to an incomplete understanding of ADRs for a given drug. Due to the small sample size of 13 fluorinated drugs and 6 matched molecular comparators and potential biases in ADR reporting, this may confound the depth of interpretation possible within this dataset.

The drugs analysed in this study were approved at separate times, for example fluoxetine in 1982 [30] and ticagrelor in 2011 [30]. This can invoke the Weber effect where a high number of ADRs are reported in the drugs in the initial years post-approval and licensing and substantial decline in reports over time [95]. This confounder may be an explanation for the difference in ADRs reported. For instance, fluoxetine, which was approved decades before ticagrelor had 37.3 ADRs/1,000,000 items and ticagrelor had 97.2 ADRs/1,000,000 items.

Furthermore, considering polypharmacology, as each drug-protein interactions had multiple IC_50_ values available the median value was used, which may not fully reflect the drugs behaviour. The lack of a complete experimental pharmacological screening data is an unavoidable limitation.

The ADR data was collected from January 2019 to May 2024, whereas the prescribing data was collected from September 2019 to August 2024 for primary care and January 2019 to June 2024 for secondary care. This unavoidable mismatch in timelines may introduce bias but can also allow for time-lag ADRs to be collected and analysed.

## Conclusion

All 13 drugs included in this study exhibited one or more ADRs that have been associated with PFAS-like side effects. However, the analysis indicates that the observed pattern of ADRs could not be attributed to either the degree of fluorination or the type of fluorine moiety present in the drugs. This finding therefore suggests that the structural similarities between these drugs and PFAS do not independently determine their ADR profile.

Instead, it was identified that the ADRs were more likely influenced by a combination of drug-specific properties, such as their pharmacokinetic and chemical properties as well as the drug classes the drug belongs to. To support this conclusion, non-fluorinated drugs within the same drug class were found to exhibit similar ADRs to the fluorinated drug, suggesting that the ADRs were attributed to the shared mechanism of action (MoA) of that drug class rather than the presence of a carbon-fluorine (C-F) bond.

Thus, the current concern that fluorinated drugs might exhibit toxicological effects similar to PFAS due to their structural similarities was unfounded in this pilot study in the UK. While fluorination may influence certain pharmacological properties of the drug, the results highlights, that the ADR patterns observed are multifactorial and are not solely determined by the presence of the C-F bond.

## Supporting information

S1 Appendix Number of ADRs reported for all SOCs and the standardised values (ADR/1,000,000 items) for the 13 fluorinated drugs. (PDF)
