# Supplementary material for: Observational suspected adverse drug reaction profiles of fluoro-pharmaceuticals and potential mimicry of per- and polyfluoroalkyl substances (PFAS) in the United Kingdom
Source: PLoS One. 2025 Sep 2;20(9):e0331286. doi: 10.1371/journal.pone.0331286 (PMC12404432; doi:10.1371/journal.pone.0331286)
Supplement: S1 Appendix — (PDF) [file pone.0331286.s001.pdf]

SUPPORTING INFORMATION

Observational suspected Adverse Drug Reaction Profiles of Fluoro-Pharmaceuticals and potential mimicry of Per- and polyfluoroalkyl Substances (PFAS) in the United Kingdom.

Banurja Balasubramaniam,[1] Alan M. Jones[1*]

[1] School of Pharmacy, School of Health Sciences, College of Medicine and Health, University of Birmingham, Edgbaston, B15 2TT, United Kingdom.

Corresponding author: a.m.jones.2@bham.ac.uk

CONTENTS

Appendix 1 **Page 2**

Appendix 1. Number of ADRs reported for all SOCs and the standardised values (ADR/1,000,000 items) for the 13 fluorinated drugs.

	Celecoxib	Flecainide	Fluoxetine	Lansoprazole	Leflunomide	Sitagliptin	Travoprost	Ezetimibe	Fluconazole	Fluocinolone	Fluticasone	Nebivolol	Ticagrelor
**Total prescriptions**	**3,513,088**	**4,451,832**	**72,023,468**	**315,918,768**	**1,879,256**	**23,128,919**	**3,332,629**	**24,453,776**	**5,290,067**	**790,377**	**46,739,343**	**6,076,689**	**5,543,070**
**Blood and lymphatic system disorders (Total ADRs)**	2	1	15	41	20	3	0	1	15	3	1	0	13
**Blood and lymphatic system disorders (ADRs/1,000,000 items)**	0.6	0.2	0.2	0.1	10.6	0.1	0	0.04	2.8	3.8	0.02	0	2.4
**Cardiac disorders (Total ADRs)**	11	62	40	126	7	6	0	10	10	2	9	11	35
**Cardiac disorders (ADRs/1,000,000 items)**	3.1	13.9	0.6	0.4	3.7	0.3	0	0.4	1.9	2.5	0.2	1.8	6.3
**Congenital, familial and genetic disorders (Total ADRs)**	0	1	9	7	2	0	0	1	0	0	1	0	0
**Congenital, familial and genetic disorders (ADRs/1,000,000 items)**	0	0.2	0.1	0.02	1.1	0	0	0.04	0	0	0.02	0	0
**Ear and labyrinth disorders (Total ADRs)**	0	1	38	27	2	0	0	5	10	1	13	3	5
**Ear and labyrinth disorders (ADRs/1,000,000 items)**	0	0.2	0.5	0.1	1.1	0	0	0.2	1.9	1.3	0.3	0.5	0.9
**Endocrine disorders (Total ADRs)**	0	0	5	24	0	0	0	1	1	3	29	0	0
**Endocrine disorders (ADRs/1,000,000 items)**	0	0	0.1	0.1	0	0	0	0.04	0.2	3.8	0.6	0	0
**Eye disorders (Total ADRs)**	4	7	81	97	12	2	28	23	33	40	41	0	5
**Eye disorders (ADRs/1,000,000 items)**	1.1	1.6	1.1	0.3	6.4	0.1	8.4	0.9	6.2	50.6	0.9	0	0.9
**Gastrointestinal disorders (Total ADRs)**	44	33	293	990	81	110	0	185	177	3	71	10	95
**Gastrointestinal disorders (ADRs/1,000,000 items)**	13.7	7.4	4.1	3.1	43.1	4.8	0	7.6	33.5	3.8	1.5	1.7	17.1
**General disorders (Total ADRs)**	33	42	301	481	126	44	3	144	177	24	103	10	51
**General disorders (ADRs/1,000,000 items)**	9.4	9.4	4.2	1.5	67.1	1.9	0.9	5.9	33.5	30.4	2.2	1.7	9.2
**Hepatobiliary disorders (Total ADRs)**	4	2	7	28	24	1	0	7	8	0	0	0	1
**Hepatobiliary disorders (ADRs/1,000,000 items)**	1.1	0.5	0.1	0.1	12.8	0.04	0	0.3	1.5	0	0	0	0.2
**Immune system disorders (Total ADRs)**	1	1	14	44	2	0	1	5	18	3	7	4	2
**Immune system disorders (ADRs/1,000,000 items)**	0.3	0.2	0.2	0.1	1.1	0	0.3	0.2	3.4	3.8	0.2	0.7	0.4
**Infections and infestations (Total ADRs)**	4	11	18	91	48	7	1	8	51	4	21	0	1
**Infections and infestations (ADRs/1,000,000 items)**	1.1	2.5	0.3	0.3	25.5	0.3	0.3	0.3	9.6	5.1	0.5	0	0.2
**Injury, poisoning and procedural complications (Total ADRs)**	5	69	144	208	31	17	4	22	31	14	84	10	28
**Injury, poisoning and procedural complications (ADRs/1,000,000 items)**	1.4	15.5	2.0	0.7	16.5	0.7	1.2	0.9	5.9	17.7	1.8	1.7	5.1
**Investigations (Total ADRs)**	4	56	93	145	32	9	4	27	38	14	13	4	21
**Investigations (ADRs/1,000,000 items)**	1.1	12.6	1.3	0.5	17.0	0.4	1.2	1.1	7.2	17.7	0.3	0.7	3.8
**Metabolism and nutrition disorders (Total ADRs)**	3	2	83	298	8	20	0	20	20	3	7	1	13
**Metabolism and nutrition disorders (ADRs/1,000,000 items)**	0.9	0.5	1.2	0.9	4.3	0.9	0	0.8	3.8	3.8	0.2	0.2	2.4
**Musculoskeletal and connective tissue disorders (Total ADRs)**	8	10	83	213	48	40	1	187	37	24	28	3	19
**Musculoskeletal and connective tissue disorders (ADRs/1,000,000 items)**	2.3	2.25	1.2	0.7	25.5	1.7	0.3	7.7	7.0	30.4	0.6	0.5	3.4
**Neoplasms benign, malignant and unspecified (Total ADRs)**	0	0	11	8	10	3	0	2	2	0	0	0	4
**Neoplasms benign, malignant and unspecified (ADRs/1,000,000 items)**	0	0	0.2	0.03	5.3	0.1	0	0.1	0.4	0	0	0	0.7
**Nervous system disorders (Total ADRs)**	20	31	437	504	41	59	4	103	88	12	110	14	51
**Nervous system disorders (ADRs/1,000,000 items)**	5.7	7.0	6.1	1.6	21.8	2.6	1.2	4.2	16.6	15.2	2.4	2.3	9.2
**Pregnancy, puerperium and perinatal conditions (Total ADRs)**	1	16	11	9	2	0	0	0	0	0	4	0	2
**Pregnancy, puerperium and perinatal conditions (ADRs/1,000,000 items)**	0.3	3.6	0.2	0.03	1.1	0	0	0	0	0	0.1	0	0.4
**Product issues (Total ADRs)**	3	8	13	27	0	4	3	7	0	8	22	4	0
**Product issues (ADRs/1,000,000 items)**	0.9	1.8	0.2	0.1	0	0.2	0.9	0.3	0	10.1	0.5	0.7	0
**Psychiatric disorders (Total ADRs)**	6	12	601	234	19	7	2	47	29	20	81	8	20
**Psychiatric disorders (ADRs/1,000,000 items)**	1.7	2.7	8.3	0.7	10.1	0.3	0.6	1.9	5.5	25.3	1.7	1.3	3.6
**Renal and urinary disorders (Total ADRs)**	5	2	17	75	23	7	2	11	11	0	7	0	18
**Renal and urinary disorders (ADRs/1,000,000 items)**	1.4	0.5	0.2	0.2	12.2	0.3	0.6	0.5	2.1	0	0.2	0	3.3

**Reproductive system and breast disorders (Total ADRs)**	0	1	95	56	0	1	0	4	38	0	6	1	1
**Reproductive system and breast disorders (ADRs/1,000,000 items)**	0	0.2	1.3	0.2	0	0.04	0	0.2	7.2	0	0.1	0.2	0.2
**Respiratory, thoracic and mediastinal disorders (Total ADRs)**	7	11	90	137	31	20	2	47	31	1	199	7	109
**Respiratory, thoracic and mediastinal disorders (ADRs/1,000,000 items)**	2.0	2.5	1.3	0.4	16.5	0.9	0.6	1.9	5.9	1.3	4.3	1.15	19.7
**Skin and subcutaneous tissue disorders (Total ADRs)**	38	19	153	501	54	47	2	65	256	40	76	6	36
**Skin and subcutaneous tissue disorders (ADRs/1,000,000 items)**	10.8	4.3	2.1	1.6	28.7	2.0	0.6	2.7	48.4	50.6	1.6	1.0	6.5
**Social circumstances (Total ADRs)**	0	0	4	4	3	0	0	0	1	0	2	0	0
**Social circumstances (ADRs/1,000,000 items)**	0	0	0.1	0.01	1.6	0	0	0	0.2	0	0.04	0	0
**Surgical and medical procedures (Total ADRs)**	0	1	3	14	7	6	0	0	2	15	2	0	0
**Surgical and medical procedures (ADRs/1,000,000 items)**	0	0.2	0.04	0.04	3.7	0.3	0	0	0.4	19.0	0.04	0	0
**Vascular disorders (Total ADRs)**	3	10	28	55	11	4	0	7	12	1	3	9	9
**Vascular disorders (ADRs/1,000,000 items)**	0.9	2.3	0.4	0.2	5.9	0.2	0	0.3	2.3	1.3	0.1	1.5	1.6
**Total (ADRs)**	210	409	2687	4444	644	417	57	939	1096	235	940	105	539
**Total (ADRs/1,000,000 items)**	59.8	91.9	37.3	14.1	342.7	18.0	17.1	38.4	207.2	297.3	20.1	17.3	97.2
